# A methodology for detecting the wound state sensing in terms of its colonization of pathogenic bacteria

**DOI:** 10.1016/j.mex.2018.11.012

**Published:** 2018-11-22

**Authors:** Jin Zhou, Danyu Yao, Zhiyong Qian, Sen Hou, Linhao Li, Yubo Fan

**Affiliations:** a Key Laboratory for Biomechanics and Mechanobiology of Ministry of Education, School of Biological Science and Medical Engineering, Beihang University, Beijing, 100083, China; b Beijing Advanced Innovation Centre for Biomedical Engineering, Beihang University, Beijing, 102402, China; c Beijing Key Laboratory of Rehabilitation Technical Aids for Old-Age Disability, National Research Center for Rehabilitation Technical Aids, Beijing, 100176, China

**Keywords:** A methodology for detecting the wound state sensing in terms of its colonization of pathogenic bacteria, Self-quenching, Carboxyfluorescein, Polydiacetylene (PDA) liposomes, Pathogenic bacteria, Fluorescence intensity

## Abstract

A methodology for wound state sensing in terms of its colonization with pathogenic bacteria such as *Staphylococcus aureus* or *Pseudomonas aeruginosa* has been developed. Here we report polydiacetylene (PDA) liposomes containing self-quenched carboxyfluorescein dye are only sensitive to toxins/enzymes secreted by Pathogenic bacteria but not by non-pathogenic species of *Escherichia coli* (DH5α). The basis of the detection assay is that at high concentration, carboxyfluorescein is non-fluorescent. Following breakdown of the bilayer of liposome containers by bacterial toxins, the dye becomes diluted and “switches on”. The methodology can be easily adapted to evaluate the release of payloads from PDA liposomes in terms of fluorescence intensity and in addition to detect the potential interaction mechanism of biomimetic bilayer and pathogenic bacteria.

• Self-quenched when encapsulated at high concentration, while fluorescence when diluted in solution
• Easy quantification by measuring fluorescence intensity
• Simple measurement procedure required (plate reading fluorimeter)

## Graphical abstract

**Figure:**
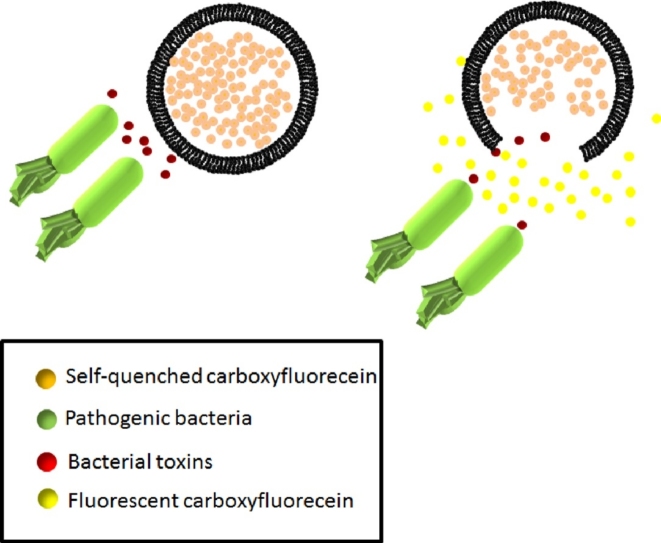

**Specifications Table**

**Table:** 

Subject area	*Select one of the following subject areas:* • *Materials Science*
More specific subject area	Nanoparticles, Drug delivery
Method name	A methodology for detecting the wound state sensing in terms of its colonization of pathogenic bacteria
Name and reference of original method	1 Zhou J, Thet Naing T, Hong S-h, Mercer-Chalmers JD, Laabei M, Young AER, et al. Development of a prototype wound dressing technology which can detect and report colonization by pathogenic bacteria. Biosensors & Bioelectronics. 2011; 30:67–72.
Resource availability	No

## Method details

### Materials and methods

#### Synthesis of PDA/phospholipid vesicles

Vesicles contained 20 mol% cholesterol (Sigma-Aldrich), 2 mol% 1,2-dimysristoyl-sn-glycero-3-phosphatidyletha-nolamine (DMPE) and varying fractions of 1,2-dimyristolyl-sn-glycero-3-phosphatidylcholine (DMPC) (Avanti Polar, USA) and 10,12-tricosadiynoic acid (TCDA) (TCI, Japan). DMPC, DMPE, cholesterol and TCDA were mixed in chloroform, dried under nitrogen. Following evaporation, PBS buffer (pH 7.4) containing drug agents was added and votexed vigorously. The suspension was then heated up at 75 °C to ensure all lipids were mixed well due to different phase transition temperatures of the different lipids. The resultant vesicle solution was then subjected to freeze-thaw cycles at least 3 times to enhance encapsulation efficiency if any drugs/dyes are encapsulated in the vesicles. The resultant vesicle solution was then extruded 20 times through pore-size polycarbonate membranes (200 nm) to obtain uniform size of vesicles and the obtained unilamellar vesicles were then dialyzed overnight through 1000 KDa membranes (Spectrum Laboratories, Inc.) to eliminate unincorporated dyes before being exposed to UV light (254 nm) for a total of 60 s from a high intensity of UV source (256 nm). The polymerized liposome solution was stored at 4 °C for further use.

#### Carboxyfluorescein fluorescence – concentration analysis

**Fig. 1:**
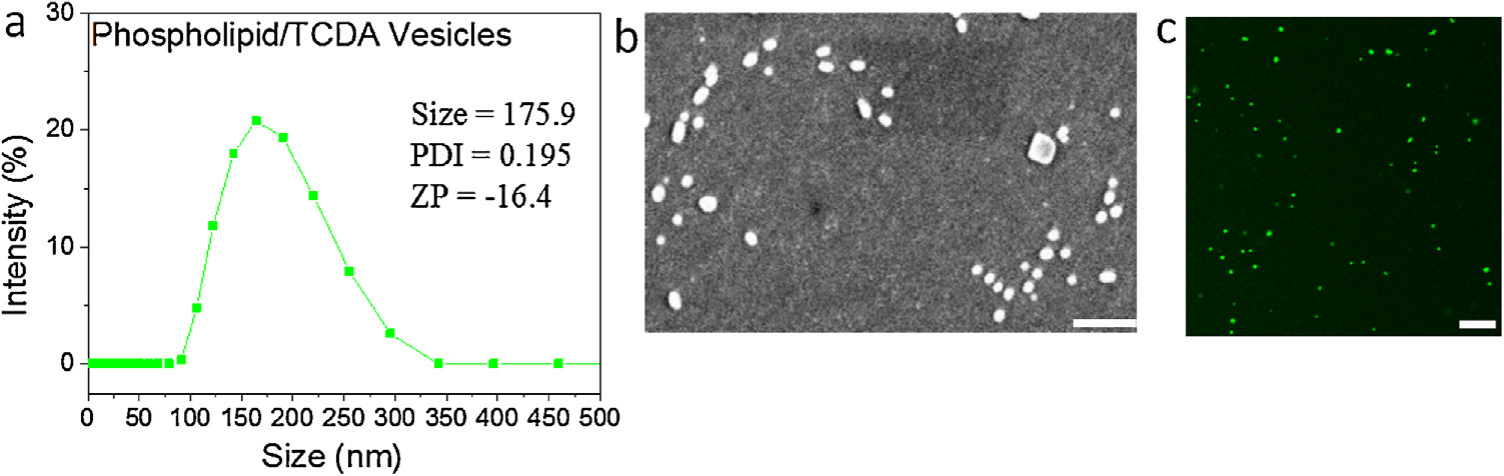
Characterization of PDA liposomes. (a) DLS studies of size, zeta potential (ZP) and polydispersity index values of PDA liposomes. (b) (c) SEM and fluorescence microscopy image indicating the morphology of PDA liposomes with the average size of ∼200 nm (scale bars: 1 1 μm). Fig. 1(a) and (b) were adopted from Ref. [5].

5(6)-Carboxyfluorescein was dissolved in HEPES buffer system at a concentration of 95 mM, which is a concentration above the threshold of concentration induced self-quenching. Serial dilutions of 5(6)-carboxyfluorescein solution were made (95 mM, 50 mM, 9 mM, 1 mM, 10^−1^ mM, 10^−2^ mM, 10^−3^ mM, 10^−4^ mM, 10^−5^ mM, 10^−6^ mM), diluted by HEPES buffer listed in Table 1. Each diluted solution (200 μL) was analyzed for fluorescence intensity with excitation at 485 nm and emission at 520 nm by using the Thermo Scientific Varioskan LUX Multimode Reader. The resultant figure was then plotted with fluorescence intensity versus various concentrations of carboxyfluorescein containing unquenched and quenched concentrations. The results are shown in Fig. 1. The HEPES buffer system for dissolving and diluting carboxyfluorescein is listed in Table 1, all the listed compounds were previously prepared in distilled water. All experiments were measured at room temperature.

**Table 1:** Composition of buffer system.

Constituent	Mass(mg)
HEPES	119.2
NaCl	29.2
NaOH	511.5
EDTA	14.2
5(6)-carboxyfluorescein	1784.6

#### Stability and sensitivity of polydiacetylene liposomes to lytic agents

To synthesize vesicles both stable for long term use (not easily to fall apart) and sensitive to toxins secreted by pathogenic bacteria, the lysis of different mole-to-mole ratios of PDA-to-synthetic liposomes by lytic toxins phospholipase A2 (PLA2) and α-hemolysin were evaluated. Triton X-100 was used as the positive control in experiments, which can cause total lysis of the bilayer membrane of vesicles. The lytic agents/enzymes and the concentration are listed in Table 2.

**Table 2:** Concentration of listed lytic agents.

Compound	Concentration
Triton X-100	1%
Phospholipase A_2_	30–120 units/mg, honey bee venom
α-Haemolysin	≥100 units/mg, *Staphylococcus aureus*

Here, the sensitivity and stability assay was based on the measurement of fluorescence intensity of carboxyfluorescein encapsulated in different PDA mol% concentration of liposomes. Following breakdown of the lipid vesicle containers by lytic agents, the fluorescent dye became diluted and “switched on”.

The first assay was based on fluorescence intensity measurement with different PDA mol% of liposomes after they are lysed by Triton X-100. The measurement was carried out in a 96-plate with excitation of 485 nm and emission of 520 nm. 100 μL vesicle solutions with various PDA mole concentrations (0%, 10%, 20%, 25%, 30%, 40%, 50%, 60%, 100%) were measured by using Thermo Scientific Varioskan LUX Multimode Reader fluorimeter for 5.5 h. The plate was then taken out of the fluorimeter and 20 μL of Triton X-100 was added into each well. The plate was put in the fluorimeter again to measure the fluorescence release overnight. The data are shown in Fig. 3a.

The second assay was conducted to evaluate the sensitivity of PDA liposomes to lytic agents. Phopholipase A2 (honey bee venom, 300–1200 units/mg, Sigma) and α-hemolysin (*Staphylococcus aureus*, ≥5000 units/mg, Sigma) as lipid-damaging enzyme and 1% Triton X-100 (Sigma) as positive control to cause the total lysis of liposomes were used for disrupting bilayer membranes of liposomes. The measurement was placed in a 100 μL liposome solution with varying PDA molar concentrations. Fluorescence was recorded as a histogram at a fixed time point, showing both the absolute value of fluorescence and increase following addition of Triton X-100 to lyse the liposomes. The data are shown in Fig. 3b.

#### Pathogenicity assay

##### Bacterial culture

Pathogenic bacteria used in the experiment were gram-positive methicillin-susceptible *S. aureus* (MRSA) and gram-negative *P. aeruginosa*, with a lab strain (non-pathogenic bacterium) of *Escherichia coli* (DH5α) as a control. The *S. aureus* and *P. aeruginosa* strains were obtained from ATCC. 10 mL LB media was used for bacterial culture for 16 h on a shaker incubator at 37 °C. 100 μL overnight culture was re-suspended in 10 mL LB media and incubated at 37 °C for 4 h. The re-suspended culture was in the exponential phase in which the virulent factors were produced by pathogenic bacteria. The procedure is called sub-culture. The sub-cultured bacteria were used in experiments unless otherwise stated, with bacterial inoculation concentration of 10^5^ CFU/mL being used.

##### Fluorescence response assay

Experiments were performed on a 96 well plate by using Thermo Scientific Varioskan LUX Multimode Reader. 200 μL of bacterial solution (10^5^ CFU/mL) in LB culture media was added in 100 μL vesicle solution. All experiments were repeated 5 times for a given set of conditions with excitation of 495 nm and emission of 520 nm. Plates were incubated at 37 °C and shaken every 30 s for 2 s prior to measurement. Fluorescence was recorded every 4 min and plotted versus time.

### Methods validation

#### Characterization of PDA liposomes

In this study, polydiacetylene liposomes with specific molar ratio of 10,12-tricosadiynoic acid (TCDA) and phospholipid composition were assembled by extrusion through 200 nm pore size of polycarbonate membranes. 20 mol% TCDA/phospholipid liposomes were synthesized in the study as a PDA liposome model. Dynamic light scattering (DLS), scanning electron microscopy and fluorescence microscopy analyses were performed to evaluate the size, size distribution and morphology of PDA liposomes (Fig. 1).

#### Liposome stability by incorporating polydiacetylene structure

We assembled a mixture of cholesterol and synthetic choline-based phospholipids that mimicked the physiologic composition of the plasmalemm [2] and enriched it with diacetylene assembly through using the thin layer evaporation (TLE) method. Unilamellar vesicles with specific molar ratio of 10, 12-tricosadiynoic acid (TCDA) and phospholipid composition were obtained by extrusion through polycarbonate membranes (200 nm pore size). The stability of the liposomes for long term use was a key concern in liposome-based sensor systems. A number of methods to stabilize liposome have been reported in the literature, including the use of sugars and photo-polymerizable lipids [3,4]. 10, 12-tricosadiynoic acid (TCDA) was used to incorporate the lipid bilayer assembly to retain their stability in the study and its sensitivity and stability were evaluated by measuring fluorescent dye (carboxyfluorescein) in terms of fluorescence intensity.

#### Carboxyfluorescein fluorescence analysis

**Fig. 2:**
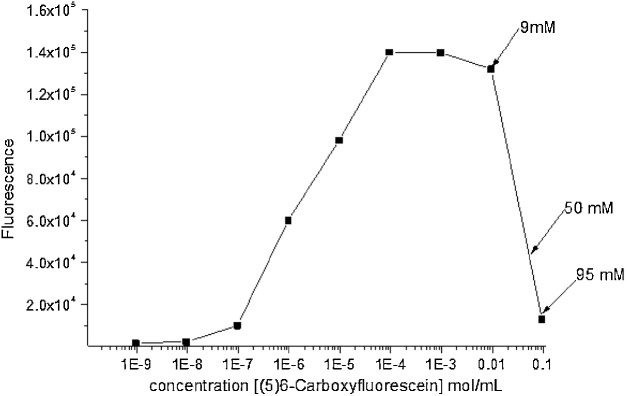
Graph showing self-quenching behavior depending upon varying concentrations. The concentration at which self-quenching occurs is when the concentration higher than 9 mM.

In order to find at which range of concentration, carboxyfluorescein begins to self-quench, the study of various concentrations of carboxyfluorescein in HEPES buffer solution versus fluorescence intensity was analyzed. Fig. 2 shows the fluorescence intensity of 5(6)-carboxyfluorescein against varying concentrations. The graph indicates the concentration at which self-quenching begins to occur. It suggests that carboxyfluorescein has dose response behavior at low concentration (<9 mM); the fluorescence intensity is dramatically decreased when the concentration is above 9 mM. It is suggested that in carboxyfluorescein solutions, the fluorescence lifetime decreased drastically as concentration changes over the narrow range 20–50 mM which is due to the energy transfer to dimers [1]. The quenched concentration of 50 mM is most commonly used in the study and as shown here up to a 10^5^ dilution will provide an increase in fluorescence.

**Fig. 3:**
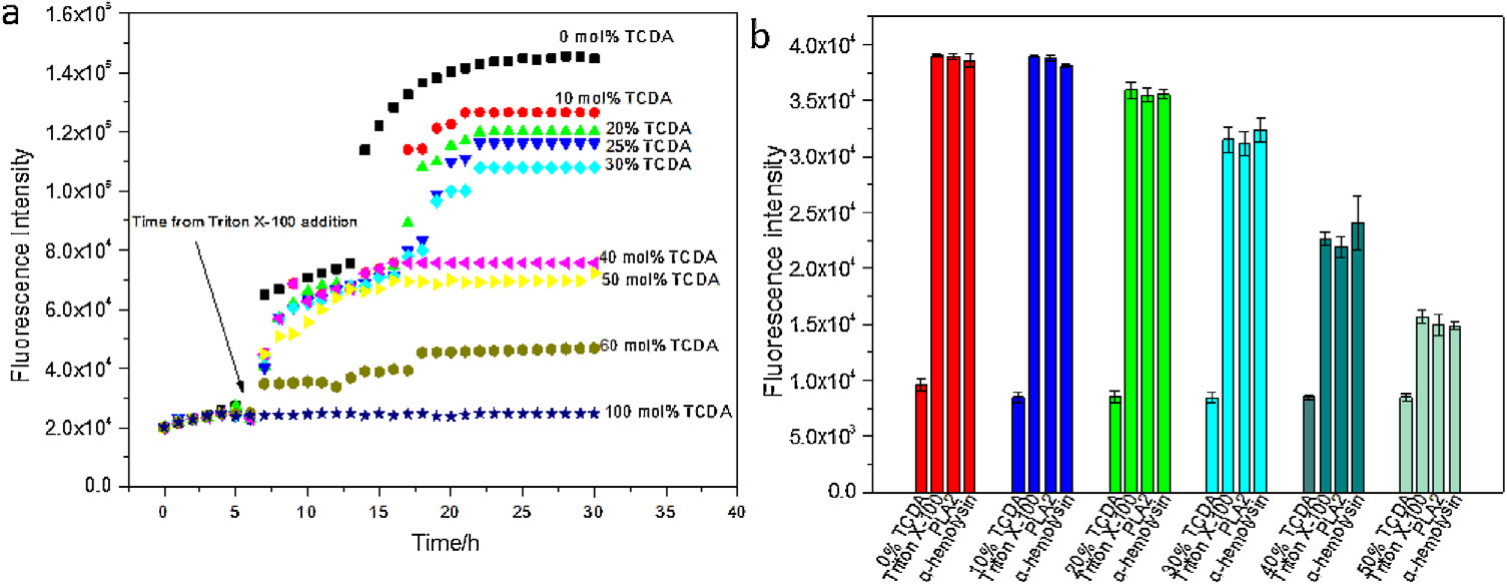
Sensitivity of PDA vesicles to different lytic agents. (a) PDA vesicle sensitivity to 1% Triton X-100 as a function of TCDA mol%. (b) PDA vesicle sensitivity to lytic toxins of phospholipase A2 (honey bee venom, 30–120 units/mg) and α-hemolysin (*Staphylococcus aureus*, ≥100 units/mg) as a function of TCDA mol%.

#### Stability and sensitivity of PDA liposomes to lytic agents

To synthesize vesicles both stable for long term use (not easily to fall apart) and sensitive to toxins secreted by pathogenic bacteria, the lysis of different mole-to-mole ratios of TCDA-to-synthetic phospholipids by phospholipase A2 (PLA2) and α-hemolysin were evaluated. 1% Triton X-100 was used as positive control to cause the total lysis of vesicles. Fig. 3a shows the plot of PDA liposome lysis by detergent: lysis of time dependence upon Triton X-100. Fig. 2a and b both show a clear decrease in fluorescence with higher concentration of polydiacetylene moiety incorporation. It is therefore concluded that an over-abundance of PDA in the bilayer membrane may lower the sensitivity of the liposomes to lytic agents. At hour-15, fluorescence intensity was increased faster between 0 mol% ∼30 mol% PDA liposomes, compared to the range of 40 mol% ∼100 mol% with gradual increase of fluorescence intensity and following a relative no release of fluorescence intensity after hour-16. This is possibly due to the relative rigid shell of liposomes in 40%, 50%, 60% and 100% and therefore small/no release of carboxyfluorescein induced by lytic agents.

#### Pathogenicity assay

**Fig. 4:**
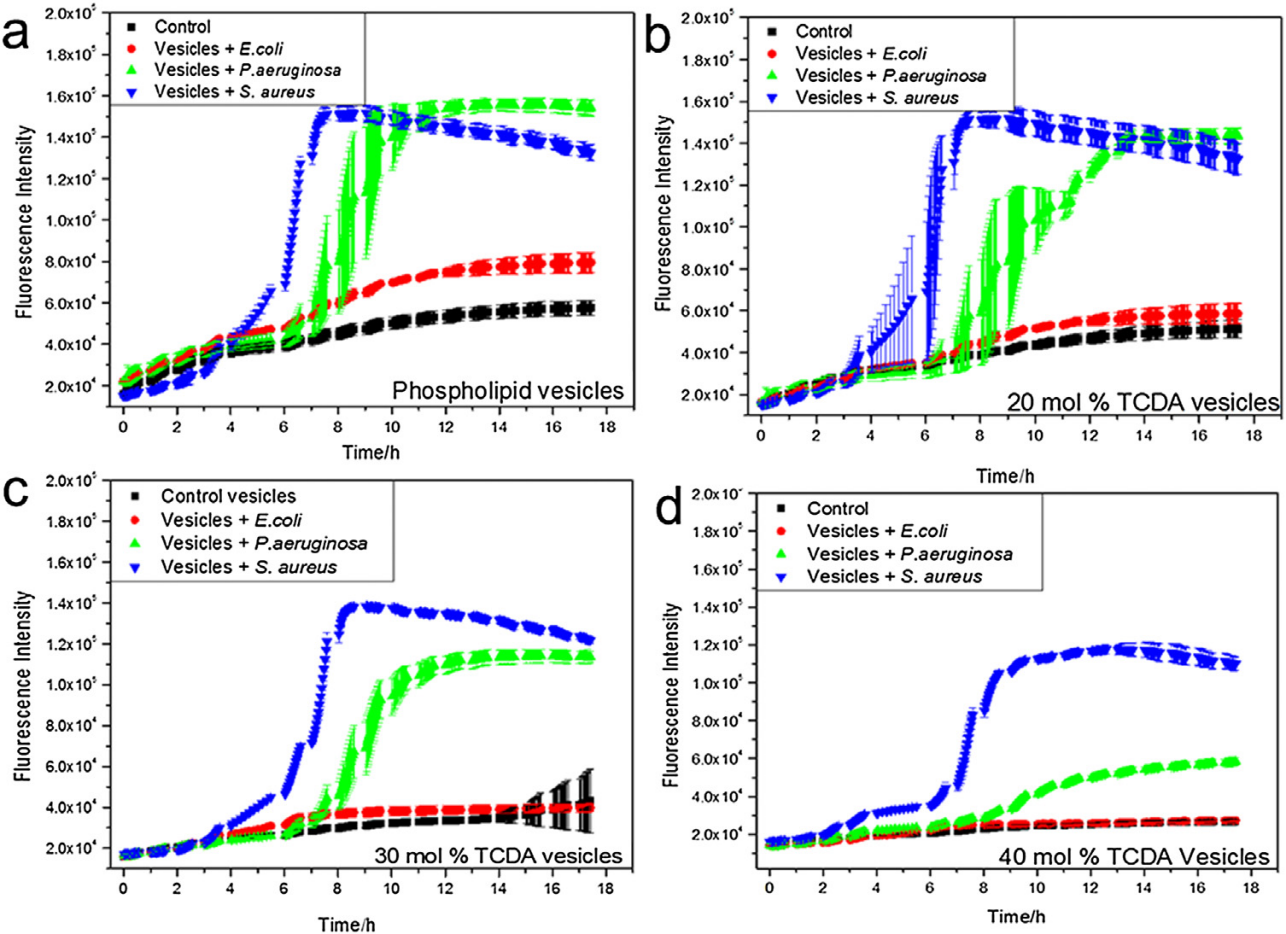
Time course of the kinetics of the interaction of bacteria with PDA vesicles. Toxins secreted by *P. aeruginosa* and *S. aureus* induced permeabilization of different concentration of PDA vesicles as revealed by the increase of fluorescence of 5(6)-carboxyfluorescein when this is released into the external solution; *E. coli* (DH5α) as control did not permeabilize vesicles. (a) phospholipid vesicles vs three strains of bacteria; (b) 20 mol% PDA vesicles vs three strains of bacteria; (c) 30 mol% PDA vesicles vs three strains of bacteria; (d) 40 mol% PDA vesicles vs three strains of bacteria.

The part of work studied the fluorescence release from vesicles with varying mol% TCDA (0, 20, 30, 40 mol%) as bacteria grew and released toxins. All experiments were suspended in buffer/bacterial growth medium. The liposomes with different PDA incorporation concentration all showed response to the two pathogenic species of bacteria as they grew (*S. aureus* and *P. aeruginosa*), whereas showed no response to the non-pathogenic strain of *E.coli* DH5α, as performed in Fig. 4. In 0% liposome assay, compared with the control group (liposomes suspended in PBS), Fig. 4a showed a slight increase in fluorescence, attributing from passive leakage of dye in liposome at 37 °C, indicating that liposomes without PDA structure would lack the relative rigidity and rendered the prototype leaky. Compared with Fig. 4a–d showed a gradual decrease in fluorescence with more mol% PDA incorporated within the lipid bilayer, suggesting that liposomes were gradually losing sensitivity to toxins produced by pathogenic strains with more PDA incorporation. Furthermore, *P. aeruginosa* grown with 40% PDA liposomes showed a 5-fold decrease in fluorescence in comparison with its growing with 20 mol % PDA liposomes, as shown in Fig. 4b and d. Interestingly, *S. aureus* seemed to be either more virulent, or secrete more enzymatic toxins during its exponential growth phase, due to the difference in mode of action on lipid bilayer of these two bacterial strains. The four graphs showed toxins produced by *S. aureus* could cause more release of fluorescence from vesicles, compared with *P. aeruginosa*.

## Conflict of interest

The authors declare no conflict of interest.

## References

[1] Chen R.F., Knutson J.R. (1988). Mechanism of fluorescence concentration quenching of carboxyfluorescein in liposomes: energy transfer to nonfluorescent dimers. Anal. Biochem..

[2] Bretscher M.S. (1972). Asymmetrical lipid bilayer structure for biological membranes. Nat.: New Biol..

[3] Yavlovich A., Singh A., Blumenthal R., Puri A. (2011). A novel class of photo-triggerable liposomes containing DPPC: DC8, 9PC as vehicles for delivery of doxorubcin to cells. Biochim. Biophys. Acta-Biomembr..

[4] Maurer N., Fenske D.B., Cullis P.R. (2001). Developments in liposomal drug delivery systems. Expert Opin. Biol. Ther..

[5] Zhou J., Yao D., Qian Z., Hou S., Li L., Jenkins A.T.A., Fan Y. (2018). Simultaneous in situ detection and inhibition of bacterial infection for accelerated wound healing. Biomaterials.

